# Real‐life challenges using personalized prognostic scoring systems in acute myeloid leukemia

**DOI:** 10.1002/cam4.5408

**Published:** 2022-11-16

**Authors:** Anne Calleja, Michael Loschi, Laurent Bailly, Adeline Morisot, Alice Marceau, Lionel Mannone, Guillaume Robert, Patrick Auberger, Claude Preudhomme, Sophie Raynaud, Fabien Subtil, Pierre Sujobert, Thomas Cluzeau

**Affiliations:** ^1^ Hematology Department Cote D'Azur University, Nice Sophia Antipolis University, CHU of Nice Nice France; ^2^ Mediterranean Center for Molecular Medecine Cote d'Azur University, INSERM U1065 Nice France; ^3^ Public Health Department, Centre Hospitalier Universitaire de Nice Cote d'Azur University Nice France; ^4^ CHRU of Lille, Hematology Laboratory Lille France; ^5^ Cote D'Azur University, Nice Sophia Antipolis University, CHU of Nice, Onco‐Hematology Laboratory Nice France; ^6^ Hospices Civils de Lyon, Hôpital Lyon Sud Service d'Hématologie Biologique Pierre‐Bénite France; ^7^ Hospices Civils de Lyon, Service de Biostatistique, CNRS, UMR 5558 Laboratoire de Biométrie et Biologie Evolutive Lyon France

**Keywords:** acute myeloid leukemia, mutations, personalized medicine, prognosis

## Abstract

Personalized medicine is a challenge for patients with acute myeloid leukemia (AML). The identification of several genetic mutations in several AML trials led to the creation of a personalized prognostic scoring algorithm known as the Knowledge Bank (KB). In this study, we assessed the prognostic value of this algorithm on a cohort of 167 real life AML patients. We compared KB predicted outcomes to real‐life outcomes. For patients younger than 60‐year‐old, OS was similar in favorable and intermediate ELN risk category. However, KB algorithm failed to predict OS for younger patients in the adverse ELN risk category and for patients older than 60 years old in the favorable ELN risk category. These discrepancies may be explained by the emergence of several new therapeutic options as well as the improvement of allogeneic stem cell transplantation (aHSCT) outcomes and supportive cares. Personalized medicine is a major challenge and predictions models are powerful tools to predict patient's outcome. However, the addition of new therapeutic options in the field of AML requires a prospective validation of these scoring systems to include recent therapeutic innovations.

The concept of personalized medicine has recently evolved with the identification of new mutations in cancer genes involved in tumor growth and survival.

In acute myeloid leukemia (AML), the relationship between the oncogenetic profile and its prognostic impact is defined by the European Leukemia Net (ELN) 2017 prognostic classification.^1^, ^2^ In 2017, *Gerstung* et al. designed a multistage AML prognostic model^3^ from 1540 patients diagnosed with AML, treated with intensive chemotherapy in three different trials. The authors combined clinical, demographic, and genomic data to design an algorithm to anticipate patients' outcome. This model predicts the remission, relapse, and mortality rates. However, this prognostic scoring system has not been prospectively validated in the era of new AML therapeutic options including targeted therapies.^4^ *Fenwarth* et al used 5‐year predictions using the model published by Gerstung et al. as a continuous variable (the KB score) in a cohort of 656 patients treated in the ALFA‐0702 trial (NCT00932412). Lower values were shown to be associated with worse prognosis and to interact with the survival benefit of HSCT in first remission. They showed that the KB score had increased predictive value compared to the ELN 2017 score for survival or progression.^5^

In order to assess the predictive value of the multistage model derived from the KB model in a single center cohort, we performed a retrospective analysis of 167 AML patients diagnosed between 2015 and 2020 at the Nice University Hospital regardless of their age and treatment. Our population was treated on a more recent time lapse than the cohorts published in other studies using the KB score and enrolled patients treated with recent therapeutic options.^6^, ^7^, ^8^ Variables were collected to calculate the score designed by Gerstung et al.^3^ We then ran the KB algorithm as published^5^ to simulate the survival of each patient from our cohort. Overall survival (OS) was calculated from the date of diagnosis until the date of death or last follow up. Eventually, we compared KB predicted outcomes to real‐life outcomes using chi‐squared test. All calculations were performed using the SPSS v22 software (IBM SPSS Statistics). Written informed consent was provided by all patients before diagnosis. The approval was registered at « Ministère de l'enseignement supérieur et de la recherche » under reference number AC‐2018‐3110.

Patients' characteristics are summarized in Table 1. Median age was 65 years (range, 19–89). One hundred and twenty five patients (76%) were diagnosed with de novo AML based on the WHO 2016 classification.^9^ Patients were classified into favorable (22%), intermediate (40%) and adverse (34%) risk according to ELN 2017 prognostic classification. Thirty‐five patients received a hypomethylating based regimen and 110 patients received intensive chemotherapy. Thirty‐one patients received targeted therapies alone or in combination. Forty patients underwent HSCT, 34 in first CR, mostly from a matched unrelated donor (23 patients), and reduced intensity conditioning (32 patients).

**TABLE 1:** Characteristics of patients

Patients characterisitcs	Number of patient (%)
Gender (M/F)	90 (54)/77 (46)
Age median at diagnosis (years)	65 (19‐90)
<60 years	60
>60 years	107
Perfomans status (ECOG)	
PS0	117
PS1	32
PS2	8
PS3	6
PS4	1
NA	3
Splenomegaly	
Present	12 (7)
Absent	141 (85)
LDH (units/L)	668 [193‐23165]
White cell count (G/L)	9,75 [0,4‐472]
Platelet count (G/L)	63 [7–725]
Periphal blood blasts (%)	22 [0–99]
Bone marrow blasts (%)	59 [10–99]
Hemoglobin (g/dL)	9,25 [3,2‐13,7]
AML type	
Pimary	125 (76)
Secondary	32 (19,5)
Therapy‐related	7 (4)
Other	2 (1,2)
ELN risk categories	156
Favorable	36(22)
Intermediaire	67 (40)
Adverse	54 (34)
Treatment	
HSCT	40 (24)
1rst CR	34
Relapse	6
Donor	
Matched related donor	9
Matched unrelated donor	23
Haploidentical donor	6
Umbilical cord blood	2
Conditionning regimen	
Myeloablative conditionning (MAC)	8
Reduced intensity conditionning (RIC)	32

Cytogenetic characteristics are available in Supplemental Table S1 and Figure S1. Karyotype evaluation was available for all patients. The most frequent abnormalities were a complex karyotype in 36 patients (21%), and del‐5/5q in 27 patients (16%). Molecular analysis, performed by NGS, for at least one mutation was available in 147 (90%) patients. Because our NGS panel included 33 of the 58 mutations from the KB model, we are missing data on the remaining 25 mutations. The most frequent mutations were *NPM1* (37 patients, 22.5%), *FLT3‐ITD* (28 patients, 17%), and *DNMT3A* (17 patients, 10.3%). We performed the KB model to predict survival and showed an AUC at 0.79 in our cohort (Supplemental Figure S2). The three‐year OS predictions by the KB model were compared to real‐life outcome (Table 2).

**TABLE 2:** Results of overall survival at 3 years, KB prediction score, and data in real life, stratified by age and ELN 2017 risk stratification

Overal survival at 3 years (%)	KB prediction	Real life	
**Total population**	46	61	*p* = 0.006
<60 years	59	68	*p* = 0.256
ELN favorable risk	73	68	*p* = 0.694
ELN intermediate risk	50	63	*p* = 0.533
ELN adverse risk	20	67	*p* = 0.003
^3^60 years	38	55	*p* = 0.014
ELN favorable risk	61	93	*p* < 0.001
ELN intermediate risk	46	54	*p* = 0.387
ELN adverse risk	20	35	*p* = 0.086

The 3‐year OS in the entire cohort was significantly higher than predicted by KB (61 vs 46%, *p* = 0.006). Because in the Gerstung cohort, the population was younger (median age of 50 years), we stratified our population according to their age and their ELN 2017 prognostic risk categories.

For patients younger than 60‐year‐old, OS was similar between algorithms predictions and real‐life with respectively 59% expected alive and 68% of the patients alive at 3 years (*p* = 0.256). However, KB predictions compared to real life were only accurate in favorable and intermediate risk categories with respectively 73% versus 68% and 50% versus 63% of patients alive at 3 years. In the adverse risk category, the KB algorithm failed to predict OS with 20% of AML patients expected to be alive according to KB predictions versus 67% of patients alive at 3 years in real‐life (*p* = 003). One explanation may be that the AML multistage prediction model was performed on patients treated between 1998 and 2006, and OS improvement in our cohort could be explained by the evolution of available AML therapies, the improvement of aHSCT and a larger access to haplo‐identical donor, as well as improvement in supportive cares (antifungal, antibiotics, intensive care). Whereas long term survival was only 30%–40% in the 2014 cohort,^10^ we noticed an improvement in the past few years with along term survival of 50%–60%.^11^

In patients older than 60‐years‐old, the KB prediction system failed to predict OS with a predicted OS of 38% patients, compared to 55% in the real‐life setting (*p* = 014). The KB algorithm had already been tested in older population and find similar results.^12^

In conclusion, we observed significant discrepancies within favorable and adverse risk categories between the KB algorithm and real‐life setting. In the favorable risk group 61% versus 93% (*p* < 0.001) and in adverse risk 20% versus 35% (*p* = 0.086) of the patients were alive at 3 years. Our findings suggest that this tool is not suitable for elderly patients at least in the favorable risk category. Failure to predict the outcome for this specific population can probably be explained by the improvement in the management of intensive chemotherapy, the availability of targeted therapies, a broader access to aHSCT and the improvement in supportive cares. Indeed older patients probably benefited the most of new targeted therapies and accessibility to HSCT.

Aside from older patients, predictions scores proved accurate in predicting OS of AML patients younger than 60 years old in favorable and intermediate ELN 2017 risk categories. However, it failed to predict OS in adverse ELN 2017 AML patients younger than 60 years old.

The KB algorithm was built on cohorts of patients younger than 60 years old treated with intensive chemotherapy. The algorithm was designed using 3 cohorts of patients treated between 1998 and 2006, with standard intensive chemotherapies, where only younger patients were eligible for aHSCT. This algorithm may therefore not be suitable to predict the outcome of older or frail patients not eligible for intensive treatment. In our cohort, older patients and adverse risk patients had a better survival rate than expected.

The significant improvements made over the past few years in supportive cares, in therapies including intensive chemotherapy, the addition of targeted therapies and the rise of reduced intensity conditioning (RIC) regimens making HSCT an option for older patients, could be some of the explanations for prediction failure in these subgroups.^13^ Indeed, several drugs like Gemtuzumab ozogamycin,^14^ CPX‐351^8^ have led to an OS improvement even in older patients. For unfit patients, several combinations with azacytidine, the current standard of care, are in development, such as AZA + venetoclax^15^ and IDH1/2 inhibitor.^16^ FLT3 inhibitors in monotherapy or in association resulted in a better OS^6^ and a higher access to aHSCT. All these therapies reduced toxicity, and increased the rate of transplanted patients in CR independently of the age. At the same time, survival rates after HSCT increased, with the use of RIC, increased donor options, improvement of supportive care and GVHD treatments,^17^, ^18^ allowing to raise the age for transplantation eligibility up to 70 years old. The strengths of our study are the balanced population ages between younger and older than 60 years treated with recent new drugs and with the improvement of aHSCT. The limitations are the small sample size, the short follow up and the monocentric study.

Personalized medicine is a major challenge for future medicine, especially with extensive molecular characterization and stratification in AML. The AML multistage prediction model is a tool allowing patients stratification. Nowadays, we are witnessing a huge development of new therapies in AML and it is necessary to prospectively validate these tools considering these new treatments.

## AUTHOR CONTRIBUTIONS

**Anne Calleja:** Data curation (lead); formal analysis (equal); validation (equal); writing – original draft (equal); writing – review and editing (equal). **Michael Loschi:** Validation (supporting); writing – review and editing (supporting). **Laurent Bailly:** Formal analysis (equal); writing – review and editing (equal). **Adeline Morisot:** Formal analysis (equal); writing – review and editing (equal). **Alice Marceau:** Data curation (equal); writing – review and editing (equal). **Lionel Mannone:** Writing – review and editing (equal). **Guillaume Robert:** Writing – review and editing (equal). **Patrick Auberger:** Writing – review and editing (equal). **Claude Preudhomme:** Data curation (equal); validation (equal); writing – review and editing (equal). **Sophie Raynaud:** Validation (equal); writing – review and editing (equal). **Fabien subtil:** Software (equal); writing – review and editing (equal). **Pierre Sujobert:** Software (equal); writing – review and editing (equal).

## CONFLICT OF INTEREST

None.

## ETHICS STATEMENT

Written informed consent was obtained for all patients and the study was local ethics committee.

## Supporting information


Figure S1

Table S1

## Data Availability

Not applicable.
