# Supplementary material for: Real‐life challenges using personalized prognostic scoring systems in acute myeloid leukemia
Source: Cancer Med. 2022 Nov 16;12(5):5656–60. doi: 10.1002/cam4.5408 (PMC10028034; doi:10.1002/cam4.5408)
Supplement: Supplementary file 1 — Figure S1 [file CAM4-12-5656-s002.pdf]

Driver mutation	yes	no
Karyotype		
Complex karyotype	36	129
fusion		
t(15;17)	9	155
inv16/t(16;16)	6	158
t(8;21)	6	158
inv3/t(3;3)	5	159
t(v;11)	2	162
t(6;9)	1	163
t(9;22)	1	163
t(9;11)	0	164
CNA[1]		
-5/5q	27	137
-17/17p/abn17p	16	148
+8	15	149
-7	11	153
-20/20q	11	153
-12/12p/abn12p	9	155
-18/18q	7	157
-Y	6	158
-7q	5	159
+13	4	160
+11/11q	4	160
+21	3	161
-4/4q/abn4q	3	161
-9q	2	162
abn7 (other)	2	162
+22	1	163
abn3q (other)	0	164
Genetics*		
NPM1	37	95
FLT3 (ITD)	28	111
DNMT3A	17	47
TP53	14	49
FLT3 (TKD)	14	117
NRAS	13	50
IDH2 (p140)	13	97
RUNX1	12	52
TET2	12	52
ASXL1	8	56
IDH1	6	104
JAK2	6	60
PTPN11	5	58
SFRS2	4	47
IDH2 (p172)	4	106
SF3B1	4	47
KRAS	3	61
EZH2	3	48
KIT	3	61
ETV6	3	48
STAG2	3	48
WT1	2	108
BCOR	2	49
CBL	2	62
U2AF1	1	40
CEBPA (mono)	1	109
GATA2	1	29
MLL	1	29
CEBPA (bi)	0	110
RAD21	0	51
FLT3 (other)	0	64
ZRSR2	0	51
PHF6	0	51
BRAF	0	12
MPL	0	51

Supplemental table 1 : cytogenetic characteristics of patients

[1] chromosomal numerical abnormalities
*Data not available **MLL2; KDM5A; MYC; CREBBP; MLL3; KDM6A; EP300; CUX1; EP301; RB1; GNAS; SF1; SF2; ATRX; PTEN; PRPF40B; U2AF2; FBXW7; SH2B3; CDKN2A; CBLB; IKZF1; MLL5; SF3A1; NF1**

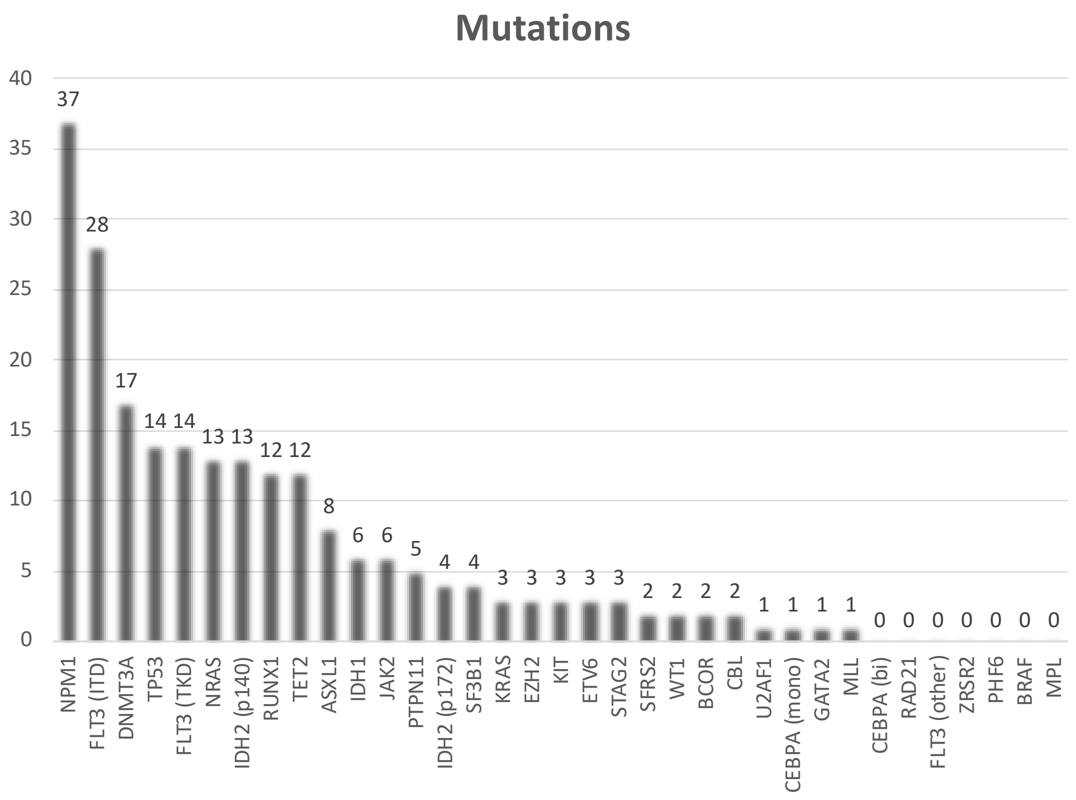

Supplemental figure 1 : number of mutations

*Data not available **MLL2; KDM5A; MYC; CREBBP; MLL3; KDM6A; EP300; CUX1; EP301; RB1; GNAS; SF1; SF2; ATRX; PTEN; PRPF40B; U2AF2; FBXW7; SH2B3; CDKN2A; CBLB; IKZF1; MLL5; SF3A1; NF1**

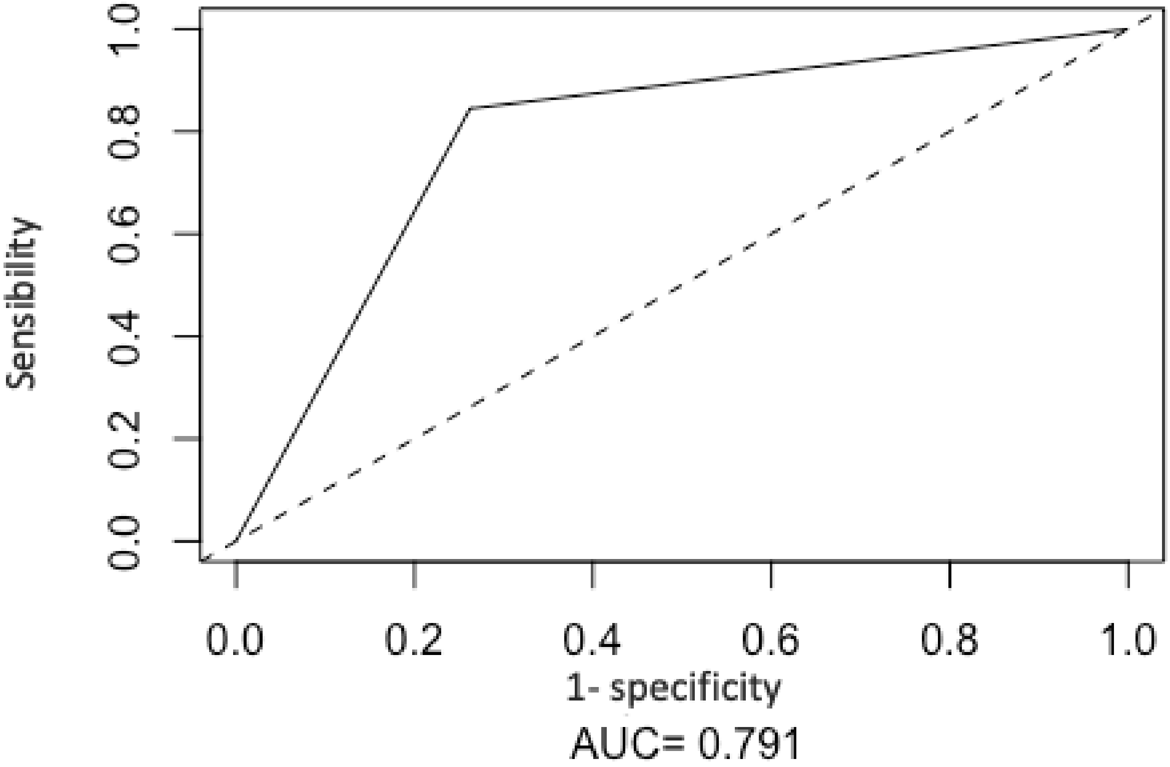

Supplemental figure 2 : ROC curve showing the performance of KB algorithm
